# Assessment of the In Vitro Biological Activities of Schiff Base-Synthesized Copper Oxide Nanoparticles as an Anti-Diabetic, Anti-Alzheimer, and Anti-Cancer Agent

**DOI:** 10.3390/pharmaceutics17020180

**Published:** 2025-02-01

**Authors:** Abdulrahman A. Almehizia, Ahmed M. Naglah, Sadeem S. Aljafen, Ashraf S. Hassan, Wael M. Aboulthana

**Affiliations:** 1 Drug Exploration and Development Chair (DEDC), Department of Pharmaceutical Chemistry, College of Pharmacy, King Saud University, P.O. Box 2457, Riyadh 11451, Saudi Arabia; mehizia@ksu.edu.sa (A.A.A.); sadeemsultan4@gmail.com (S.S.A.); 2 Organometallic and Organometalloid Chemistry Department, National Research Centre, Dokki, Cairo 12622, Egypt; 3 Biochemistry Department, Biotechnology Research Institute, National Research Centre, Dokki, Cairo 12622, Egypt; wm.kamel@nrc.sci.eg

**Keywords:** nanotechnology, copper oxide nanoparticles, transmission electron microscope (TEM), anti-diabetic and anti-Alzheimer activities, cyclooxygenases, enzymatic assay

## Abstract

**Background/Objectives:** Numerous diseases such as diabetes, Alzheimer’s disease, and cancer have spread in the whole world, especially in the Arab world. Also, various applications of Schiff-base functionalized nanoparticles and copper oxide nanoparticles (CuO-NPs) such as therapeutic applications have been discovered. Thus, the current research highlights (i) the synthesis of copper oxide nanoparticles (CuO-NPs) produced with a Schiff base (SB) serving as a capping agent during their synthesis and (ii) assessment of the in vitro biological activities of Schiff base-synthesized copper oxide nanoparticles (SB-CuO-NPs) and a Schiff base (SB). **Methods:** SB-CuO-NPs were characterized using ultraviolet-visible (UV-Vis) spectroscopy, zeta potential, DLS analysis, and transmission electron microscope (TEM). It also focuses on assessing the in vitro biological applications and activities, including antioxidant, scavenging, anti-diabetic, anti-Alzheimer, anti-arthritic, anti-inflammatory, cytotoxic activities, and enzymes inhibitory potential, of Schiff base-synthesized copper oxide nanoparticles (SB-CuO-NPs) and a Schiff base (SB) using methods described in the literature. **Results:** The results of the biological activities of the SB-CuO-NPs were compared with those of the SB. The SB-CuO-NPs demonstrated superior in vitro biological activities when compared to the SB from which they were produced. **Conclusions:** The results of this investigation concluded that the CuO-NPs, synthesized with the SB serving as an alternative capping agent, exhibited enhanced biological efficacy relative to the original SB. In the future, the biological efficiency of SB-CuO-NPs against diabetes, Alzheimer’s, and cancer diseases will be assessed in experimental animals (in vivo).

## 1. Introduction

Recently, numerous diseases such as diabetes, Alzheimer’s disease, and cancer have spread in the Arab world and even in the whole world, including countries like Egypt and Saudi Arabia [1,2]. It has become necessary to utilize novel and innovative strategies, such as nanotechnology, to develop new compounds with therapeutic applications against these diseases [3,4,5].

The primary method for preventing and treating chronic disorders caused by excessive lipid oxidation and inflammation is the use of exogenous antioxidants [6]. The pathophysiology of diabetes, Alzheimer’s, and arthritic diseases is caused by degenerative processes that result in inflammation, neural damage, and protein misfolding, which are brought on by oxidative stress and excessive lipid oxidation [7].

A metabolic disease called diabetes mellitus is typified by elevated glucose levels. Blood glucose levels are regulated by the enzymes α-amylase and α-glucosidase. The former breaks down carbs into disaccharide molecules, while the latter turns those disaccharides into monosaccharide molecules [8]. In order to quantify the anti-diabetic efficacy of several compounds, their inhibitory action against both enzymes has been evaluated. The results were then compared with acarbose, a conventional drug [9].

Alzheimer’s disease is a neurodegenerative condition triggered by oxidative stress, marked by a decline in cognitive abilities and memory, as well as disruptions in logical reasoning and emotional stability. Acetylcholinesterase (AChE) is a dependable enzyme that plays a crucial role in the hydrolysis of acetylcholine within cholinergic synapses linked to β-amyloid plaques. Consequently, the inhibition of AChE represents a therapeutic approach for managing Alzheimer’s disease [10].

Cancer encompasses a variety of diseases marked by the presence of malignant tumors that result from the unregulated proliferation of cells, which can invade and damage adjacent tissues, ultimately leading to mortality if not managed effectively [11]. The cancer therapy strategy depends on various factors. The patient’s health state is an important factor in determining this strategy [12]. Recently, cancer nanovaccines use nanoparticles (NPs) for immunotherapy. Nanovaccines are one of novel and effective strategies for cancer therapy [13].

Arthritis is classified as an inflammatory disease that arises from the denaturation of proteins and heightened activity of the proteinase enzyme. Consequently, the inhibition of this enzyme is regarded as a promising approach for the treatment of arthritis [14].

Nanotechnology is a promising field applied in numerous sciences, e.g., chemistry, engineering, and medicine. Materials with dimensions between 1 and 100 nm are the subject of nanotechnology field interest. In general, materials in the nanoscale exhibit properties different from larger-sized materials [15]. Abid et al. prepared green-synthesized Fe₃O₄NPs from Cascabela Thevetia and evaluated their in vitro and in vivo properties. The results showed that these Fe₃O₄NPs possess hemolytic, anti-inflammatory, and anti-diabetic potential [16]. Nowadays, nanoparticles are used in cancer treatment, particularly skin cancer, because they possess advantages that can overcome traditional and standard therapy [17].

The Schiff bases, imine group (>C=N−), possess biological importance and have various applications since their discovery [18,19,20]. Schiff bases are used as ligands with different elements to form metal complexes [21,22]. A literature survey has shown that metal complexes have biological importance [23,24,25]. Recently, Schiff bases have been utilized in nanotechnology [26]. The imine group of Schiff base ligands stabilizes the nanoparticles [27]. Various applications of Schiff-base functionalized nanoparticles, such as catalytic and biological behaviors, have been found [28].

Bioactive compounds typically possess a high molecular weight, which can hinder their absorption across cellular membranes, thereby reducing their bioavailability and overall effectiveness. To address these challenges, nanotechnology is employed to enhance bioavailability, improve efficiency, increase stability and solubility, minimize toxic effects, and facilitate targeted release at the desired site of action [29]. The integration of metal nanoparticles (M-NPs) is regarded as a highly promising approach to address the challenges associated with the inadequate solubility, distribution, and absorption characteristics of organic compounds [30].

Copper oxide nanoparticles (CuO-NPs) usage has significantly expanded in several applications such as water treatment [31], biomedicines [32], and bioengineering [33] due to their flexible properties. CuO-NPs have shown significant antimicrobial, antioxidant [34], and anticancer [35] properties. Moreover, CuO-NPs have an impact on plant growth [36]. Additionally, CuO-NPs have lower toxicity compared to silver (Ag), gold (Au), and zinc oxide nanoparticles (ZnO-NPs) [37].

Based on the information above and in continuation of our targets [38,39,40,41], this study was conducted to explore the in vitro biological efficacy of copper oxide nanoparticles (CuO-NPs) synthesized with the aid of Schiff base (SB) serving as a capping agent during the synthesis process.

## 2. Materials and Methods

### 2.1. Preparation of SB and SB-CuO-NPs

Schiff base (SB) [42] and SB-CuO-NPs [43] were prepared according to the methods described in the literature (See Supplementary Materials).

### 2.2. Characterization of SB-CuO-NPs

The SB-CuO-NPs were characterized using UV-Vis spectroscopy, zeta potential, DLS analysis, and TEM according to the reported methods [44,45,46] (See Supplementary Materials).

### 2.3. In Vitro Biological Activities

The in vitro biological applications of the SB and SB-CuO-NPs, including antioxidant [47,48], scavenging [49,50,51], anti-diabetic [52,53], anti-Alzheimer [54], anti-arthritic [55,56,57], anti-inflammatory [58,59], cytotoxic effects [60,61], and enzyme inhibition [62,63], were assessed using methods described in the literature (See Supplementary Materials).

Statistical analysis: The details are in the Supplementary Materials.

## 3. Results and Discussion

### 3.1. Preparation of the Schiff Base (SB)

**Scheme 1:**
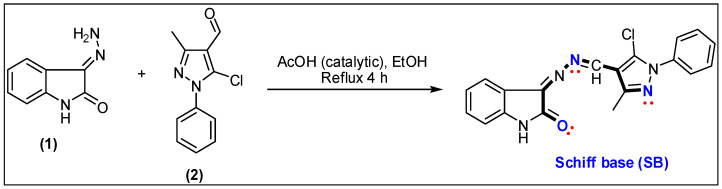
Synthesis of SB.

The Schiff base (SB) was prepared according to the reported method [42] by reacting 3-hydrazonoindolin-2-one (1) with 5-chloro-3-methyl-1-phenyl-1*H*-pyrazole-4-carbaldehyde (2) (Scheme 1). The characterization was also described in our previous work [42]. The data and chart of the ^1^H-NMR spectrum of SB were provided in the Supplementary Materials.

The Schiff base was formed by acid-catalyzed (water elimination). The ^1^H-NMR spectrum of SB exhibits the presence of the azomethine proton (-CH=N-) at δ 8.65 ppm. This signal confirms the Schiff base formation.

### 3.2. Preparation and Characterization of the Schiff Base-Copper Oxide Nanoparticles (SB-CuO-NPs)

**Figure 1:**
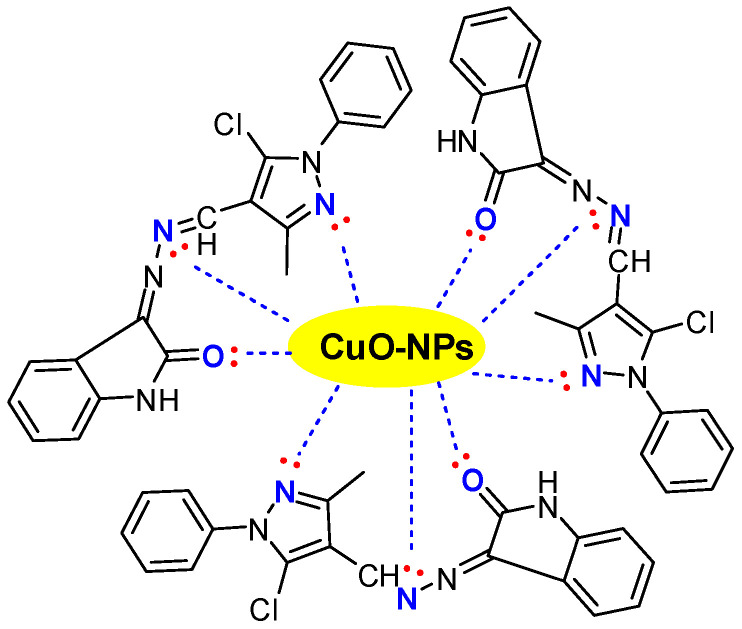
Exhibits visualization of SB-CuO-NPs.

The synthesis of copper oxide nanoparticles (CuO-NPs) produced with a Schiff base (SB) serves as a capping agent during their synthesis. Figure 1 exhibits visualization of bonding between SB ligand and copper oxide nanoparticles. This visualization was based on the azomethine group and the electron pairs of the nitrogen and oxygen atoms, which stabilize the nanoparticles through bond formation [64,65].

**Figure 2:**
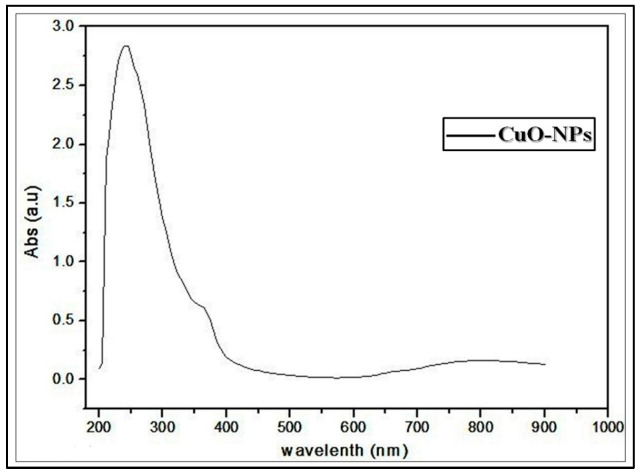
UV-VIS spectroscopy of the SB-CuO-NPs.

Ultraviolet-visible (UV-Vis) spectroscopy serves as a crucial method for assessing the morphology and stability of nanoparticles. In this study, a wavelength range of 280 to 400 nm was employed to characterize SB-CuO-NPs. The absorbance of the reaction mixture was recorded after a 24-h period, during which it was maintained at a temperature of 25 °C in a dark environment. The SB-CuO-NPs, as illustrated in Figure 2, exhibit a peak absorbance at approximately 296 nm, which is attributed to the surface plasmon absorption characteristic of nanosized cupric oxide particles, as noted by Dobrucka [66]. The UV-Vis spectra for all samples reveal a prominent absorption peak at 275 nm, linked to the surface plasmon resonance of the CuO nanoparticles. This phenomenon arises from the collective oscillation of electrons within the free conduction band, stimulated by incoming electromagnetic radiation, as discussed by Naika et al. [67]. Furthermore, the absorption intensity of the samples demonstrates an increase corresponding to higher copper concentrations, indicating a greater formation of nanoparticles resulting from the reduction in copper ions, as reported by Kumar et al. (2020) [68].

**Figure 3:**
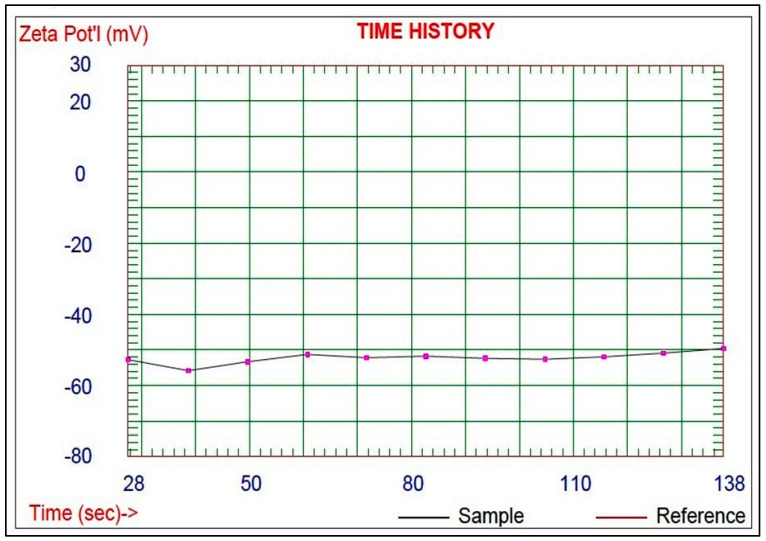
Zeta potential of the SB-CuO-NPs.

The SB-CuO-NPs exhibit a zeta potential of 63.2 mV and possess a particle size of approximately 100 nm, indicating remarkable stability, as illustrated in Figure 3. This finding aligns with the assertions made by Kolahalam et al. [69], who suggested that a high zeta potential of more than 30 mV, whether the charge is positive or negative, is indicative of superior physical colloidal stability, likely due to the electrostatic repulsion among particles. Conversely, a reduced zeta potential may suggest the potential aggregation of particles.

**Figure 4:**
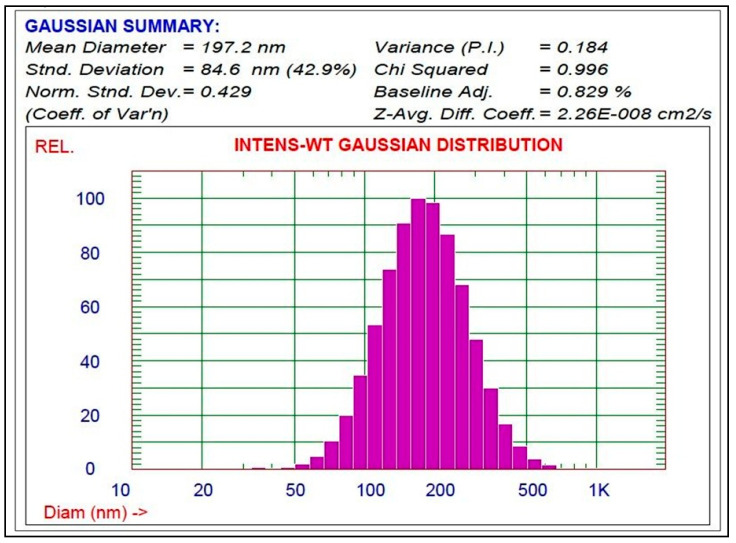
Dynamic light scattering (DLS) of the SB-CuO-NPs.

The data obtained from the dynamic light scattering (DLS) analysis indicate that the particle size distribution of the SB-CuO-NPs predominantly centers around a diameter of approximately 200 nm, as illustrated in Figure 4. This observation suggests that the particles exhibit a larger and more polydisperse nature when compared to the measurements derived from UV spectroscopy and zeta potential assessments. This finding is consistent with the work of Nzilu et al. [70], who noted that the diameter of biosynthesized SB-CuO-NPs typically falls within the range of 0 to 200 nm, characterized by a significant population. Furthermore, the polydispersity index suggests that the size distribution of the particles is monodispersed, which may be attributed to agglomeration or aggregation occurring during the biosynthesis process.

**Figure 5:**
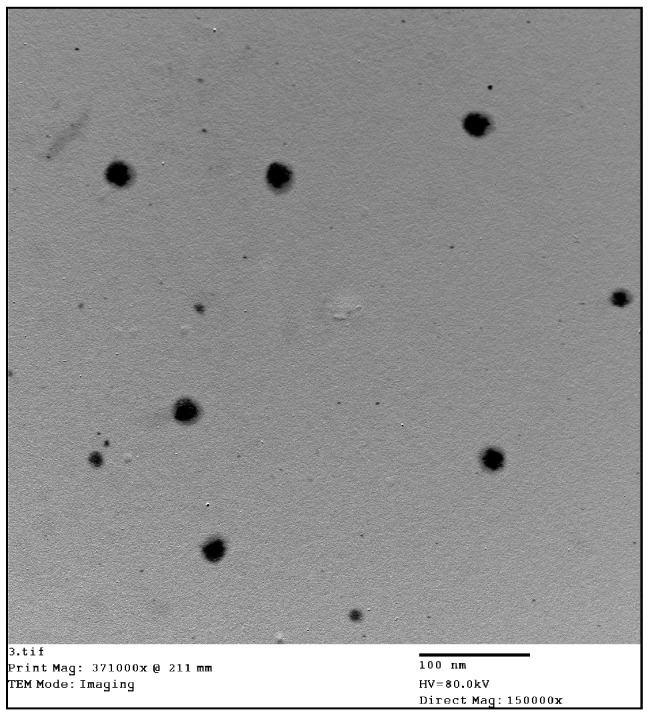
TEM of the SB-CuO-NPs.

Transmission electron microscope (TEM) is a technique used for the characterization of the size, shape, and distribution of nanomaterials. The morphology of SB-CuO-NPs was examined by the TEM. Figure 5 shows the TEM image of the SB-CuO-NPs. The length of 100 nm (ــــــــــــــــــــ) was represented as a key in Figure 5. By measuring the SB-CuO-NPs particles according to the 100 nm length, we found that the SB-CuO-NPs have an average width of approximately 17 nm and a round or spherical shape. Also, the distribution appears to be regular.

### 3.3. In Vitro Biological Activities

#### 3.3.1. Antioxidant and Scavenging Activity

One of the fundamental investigations in the field of nanoscience and technology involves evaluating the antioxidant properties of nanomaterials. As indicated in Table 1, the introduction of SB-CuO-NPs resulted in an enhancement of antioxidant activity, as evidenced by the TAC (76.53 ± 0.22 mg gallic acid/g) and IRP (58.78 ± 0.22 µg/mL). These values surpass those of the Schiff base (SB), which recorded TAC and IRP values of 65.41 ± 0.19 mg gallic acid/g and 47.66 ± 0.19 µg/mL, respectively; however, they remain lower than those of ascorbic acid, which serves as the standard reference. This finding is consistent with the research conducted by Djamila et al. [71], which indicated that antioxidant activity is positively correlated with the concentration of CuO-NPs.

The FRAP assay is recognized for its ability to assess antioxidant activity through the reduction in Fe^3+^ ions to Fe^2+^ ions by the substance under investigation. This reduction in ferric iron results in the formation of a blue color, which can be quantitatively analyzed using colorimetric methods at a wavelength of 594 nm [72].

During the current study, the free radical scavenging capacity of SB-CuO-NPs, SB, and ascorbic acid (positive control) was evaluated using the DPPH, ABTS, and NO radical methods. The scavenging activity is used to predict antioxidant efficacy, a mechanism in which antioxidants inhibit lipid oxidation. The lower IC_50_ value indicates a stronger ability of the SB-CuO-NPs to act as radical scavengers, whereas the higher IC_50_ value indicates a lower scavenging activity [73]. The IC_50_ value is a parameter indicative of the inhibitory concentration required to decrease the radical by 50%. The lower the IC_50_ value, the greater the antioxidant activity [74].

Results in Table 1 show that the SB-CuO-NPs possess strong inhibitory activity against DPPH, ABTS, and NO radicals with IC_50_ values of 5.06 ± 0.01, 4.45 ± 0.05, and 5.99 ± 0.03 μg/mL, respectively. These results were compared to ascorbic acid, a conventional antioxidative agent that possessed potency with IC_50_ values of 4.50 ± 0.03, 3.99 ± 0.04, and 5.24 ± 0.02 μg/mL, respectively. The SB-CuO-NPs belong to the antioxidants, which have been used to mitigate the negative effects of ROS-induced inflammation, especially to reduce mutations and damage caused to host tissue [75].

#### 3.3.2. Anti-Diabetic and Anti-Alzheimer’s Activities

**Figure 6:**
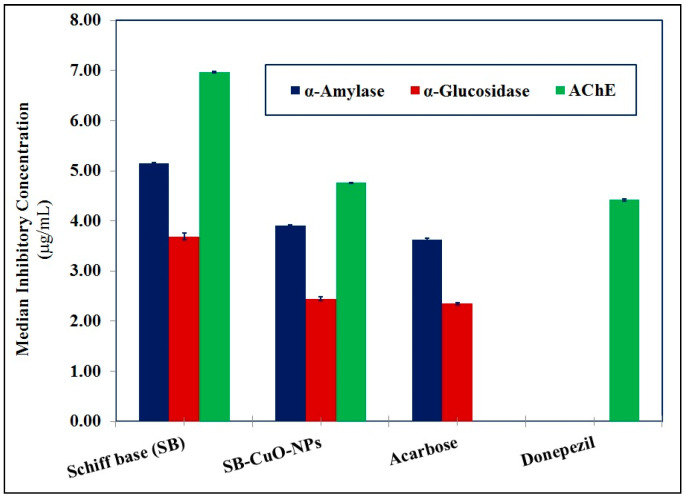
IC_50_ values of the SB-CuO-NPs, SB, and standard references against α-amylase, α-glucosidase, and acetylcholinesterase (AChE) enzymes.

The assessment of α-amylase and α-glucosidase inhibition is an indicator of the anti-diabetic efficacy of compounds [76]. In the current investigation, it was noted that the SB-CuO-NPs exhibited an enhanced inhibitory effect on the activity of α-amylase (Inhibition = 51.03 ± 0.18%; IC_50_ = 3.90 ± 0.02 µg/mL) and α-glucosidase (Inhibition = 42.82 ± 0.19%; IC_50_ = 2.45 ± 0.04 µg/mL) when compared to the original synthetic derivative. In contrast, acarbose demonstrated a similar concentration-dependent inhibitory effect on α-amylase (Inhibition = 54.96 ± 0.10%; IC_50_ = 3.62 ± 0.03 µg/mL) and α-glucosidase (Inhibition = 44.71 ± 0.10%; IC_50_ = 2.35 ± 0.02 µg/mL) as presented in Table 2 and Figure 6. These results are consistent with the findings of Kainat et al. [77], who highlighted that hydrolytic enzymes, particularly α-amylase, are the most vulnerable diabetic enzymes to interactions with M-NPs.

Alzheimer’s disease therapy is based on acetylcholinesterase (AChE) enzyme inhibition. Hence, compounds that possess the ability and potential for AChE enzyme inhibition, may act as Alzheimer’s disease drugs [78]. In the current study, it was noted that SB-CuO-NPs exhibited a heightened inhibitory effect on the activity of the AChE enzyme, with an inhibition rate of 47.83 ± 0.21% and an IC_50_ value of 4.76 ± 0.01 µg/mL. This effect was found to be more pronounced than that of the SB and the standard reference, Donepezil, which demonstrated an inhibition of 51.42 ± 0.12% with an IC_50_ of 4.42 ± 0.02 µg/mL, as detailed in Table 2 and Figure 6. This phenomenon may be linked to the deterioration of aromatic amino acid residues and/or a rise in the quantity of water molecules within the hydration shell surrounding the protein component, resulting in a modification of the refractive index [79].

#### 3.3.3. Anti-Arthritic and Anti-Inflammatory Activities

The arthritic response is attributed to the denaturation of proteins and the functioning of the proteinase enzyme. The capacity of the substance to prevent protein denaturation and inhibit the proteinase enzyme suggests its potential role in exhibiting anti-inflammatory properties. Consequently, the evaluation of anti-arthritic activity was conducted by analyzing these specific parameters [80]. The present investigation revealed that the SB-CuO-NPs demonstrated a significant inhibitory effect on protein denaturation, achieving an inhibition percentage of 38.27 ± 0.17. Additionally, these nanoparticles inhibited the activity of the proteinase enzyme, with an inhibition rate of 35.57 ± 0.17% and an IC_50_ value of 7.13 ± 0.03 µg/mL. In comparison, the standard reference, Diclofenac Sodium, exhibited inhibition percentages of 41.14 ± 0.09 for protein denaturation and 38.44 ± 0.09 for proteinase enzyme activity, as detailed in Table 3.

**Figure 7:**
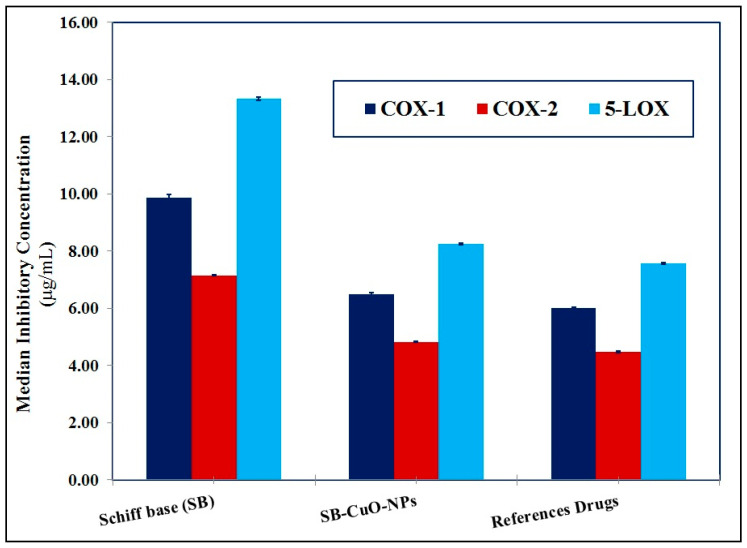
IC_50_ values of the SB-CuO-NPs, SB, and standard references against COX-1, COX-2, and 5-LOX enzymes.

Lipoxygenase (5-LOX) and cyclooxygenase (COX) are prominent pro-inflammatory enzymes commonly utilized to assess the anti-inflammatory properties of M-NPs, as noted by El Feky and El-Rashedy [81]. COX exists in two isoforms, COX-1 and COX-2, which are distinguished by their intracellular distributions and structural compositions, yet both isoforms fulfill identical roles in physiological processes, according to Mukhopadhyay et al. [82]. The SB-CuO-NPs exhibited an enhanced inhibitory effect on the activities of COX-1 (Inhib. = 48.02 ± 0.23%; IC_50_ = 6.49 ± 0.07 µg/mL), COX-2 (Inhib. = 50.27 ± 0.23%; IC_50_= 4.83 ± 0.02 µg/mL), and 5-LOX enzymes (Inhib. = 43.12 ± 0.23%; IC_50_ = 8.25 ± 0.03 µg/mL) when compared to the SB and the respective standards (Table 3 and Figure 7).

The standard reference demonstrates inhibitory activities of COX-1(indomethacin) (Inhib. = 51.89 ± 0.13%; IC_50_ = 6.01 ± 0.02 µg/mL), COX-2 (indomethacin) (Inhib. = 54.14 ± 0.13%; IC_50_ = 4.47 ± 0.03 µg/mL), and 5-LOX (Zileuton) enzymes (Inhib. = 46.99 ± 0.13%; IC_50_ = 7.57 ± 0.03 µg/mL), as detailed in Table 3.

This aligns with the findings of Rehman et al. [83], who suggested that M-NPs demonstrate anti-inflammatory properties by inhibiting COX-1, COX-2, and 5-LOX in a concentration-dependent manner. Additionally, the inhibition of these enzymes may result from their denaturation on the surface of M-NPs and/or the effects of charge. Cha et al. [84] further noted that smaller M-NPs are likely to exhibit the most pronounced inhibitory effects. The degree of inhibition is influenced by the morphology of the biosynthesized M-NPs, with an increase in enzyme activity inhibition observed as the concentrations of nanopyramids and nanoplates rise, whereas the enzyme activity remained largely unchanged across all concentrations of nanospheres.

Furthermore, statistical analysis was performed, and the data were documented in Table S1 (See Supplementary Materials). The results indicate that measurements of in vitro biological activities were positively correlated.

#### 3.3.4. Cytotoxic Activity and Enzymatic Assay

**Figure 8:**
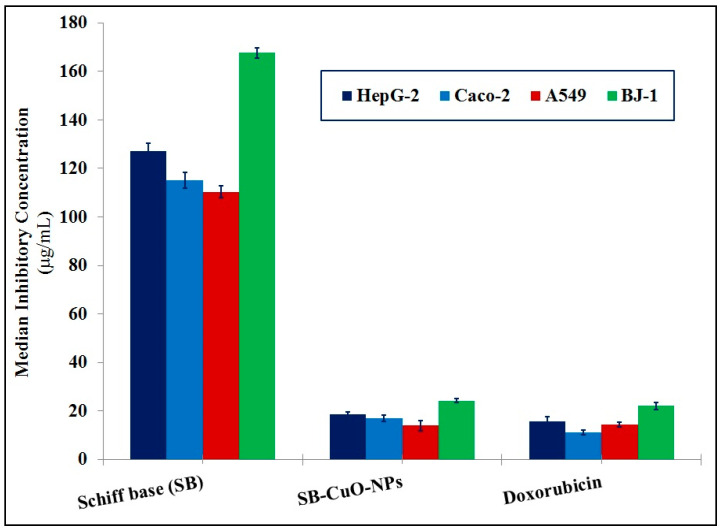
IC_50_ values of the SB-CuO-NPs, SB, and doxorubicin against (HepG-2), colon (Caco-2), lung (A549) cancer cell lines.

In the current investigation, the cytotoxic effects observed against the examined cell lines are detailed in Supplementary Tables S2–S5. It was observed that the SB-CuO-NPs exhibited enhanced cytotoxic activity against HepG-2 (IC_50_ = 18.68 ± 0.83 µg/mL), Caco-2 (IC_50_ = 17.08 ± 1.30 µg/mL), A549 (IC_50_ = 13.87 ± 2.25 µg/mL), and BJ-1 cells (IC_50_ = 24.23 ± 0.76 µg/mL) when compared to the SB, which showed IC_50_ values of 127.16 ± 3.13, 115.16 ± 3.25, 110.37 ± 2.49, and 167.65 ± 2.22 µg/mL, respectively (Table 4 and Figure 8).

The results of the present study align with the findings of Tripathi et al. [85], which indicated that the synthesized M-NPs promote apoptotic death in cancer cells while simultaneously inhibiting cell proliferation and survival, contingent upon their concentration and internalization via endocytosis. Additionally, the small size and large surface area of the M-NPs enable them to obstruct antioxidant molecules, resulting in the production of elevated levels of reactive oxygen species (ROS) within tumor tissues, thereby inducing oxidative stress. This oxidative stress can trigger the infiltration of cells, the activation of apoptotic mechanisms, and the initiation of inflammatory pathways, as noted by Li et al. [86].

The prevalent strategies for anti-cancer therapy focus on enhancing pro-apoptotic molecules while inhibiting those that are anti-apoptotic [87]. According to the findings of Naglah et al. [9], the derivative exhibiting cytotoxic properties was found to elevate the activity of the caspase-3 enzyme, concurrently reducing the levels of Bcl-2 in cancer cells that were treated, in contrast to those that remained untreated.

**Figure 9:**
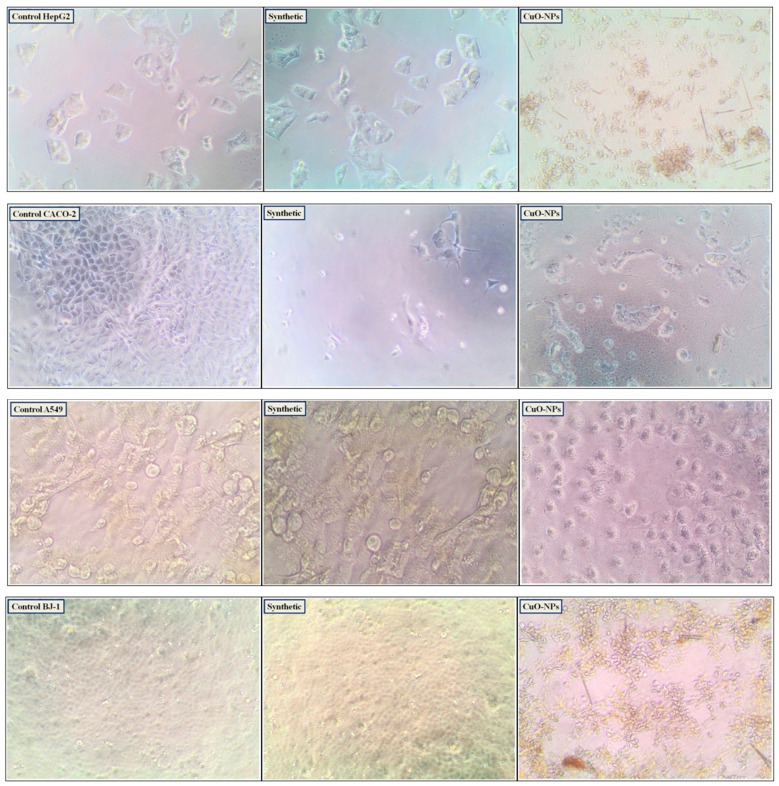
Illustrates the human cancer cell lines (HepG-2, Caco-2, and A549) with the magnification power 40× and the normal cell line (BJ-1) with 16× as controls, treated with the IC_50_ concentration of the SB and the SB-CuO-NPs.

Following the treatment of HepG-2, Caco-2, A549, and BJ-1 cells with the SB-CuO-NPs at their respective IC_50_ values, as illustrated in Figure 9 and detailed in Table 5, an assessment of the activities of the caspase-3 and Bcl-2 enzymes was conducted in the treated cells. The results indicated that the highest levels of caspase-3 activity were recorded in HepG-2 (291.00 ± 1.34 Pg/mL), Caco-2 (252.31 ± 0.30 Pg/mL), A549 (236.30 ± 0.22 Pg/mL), and BJ-1 cells (191.03 ± 0.22 Pg/mL) following exposure to the SB-CuO-NPs. Conversely, the lowest activities of the Bcl-2 enzyme were noted in these treated cells, with values of 4.17 ± 0.05, 2.14 ± 0.01, 4.72 ± 0.02, and 2.85 ± 0.02 ng/mL, respectively. According to the findings of Khaled et al. [88], the biosynthesized M-NPs demonstrate a significant antitumor effect on cancer cells by enhancing the activity of the caspase-3 enzyme while simultaneously reducing the levels of Bcl-2. This mechanism ultimately triggers apoptosis. Additionally, the increased activity of caspase-3 results in a decrease in the expression of cyclin D1 and CDK-2 mRNA, thereby promoting the arrest of the G1 phase of the cell cycle. Othman et al. [89] further noted that the biosynthesized M-NPs facilitate apoptosis by inhibiting the expression of the PI3K/AKT and Ras/Raf/ERK protein signaling pathways.

## 4. Conclusions

In this work, SB-CuO-NPs were prepared and characterized using UV-Vis spectroscopy, zeta potential, DLS analysis, and TEM. The in vitro biological applications of SB-CuO-NPs and SB were assessed and compared to the corresponding references. The in vitro biological applications results demonstrated that SB-CuO-NPs possess high antioxidant activity (TAC = 76.53 ± 0.22 mg gallic acid/g and IRP= 58.78 ± 0.22 µg/mL) and radicals scavenging (DPPH = 5.06 ± 0.01 μg/mL, ABTS = 4.45 ± 0.05 μg/mL, and NO radicals = 5.99 ± 0.03 μg/mL). Anti-diabetic results show α-amylase inhibition % of 51.03 ± 0.18% with IC_50_ = 3.90 ± 0.02 µg/mL and α-glucosidase inhibition % of 42.82 ± 0.19% with IC_50_ = 2.45 ± 0.04 µg/mL. Also, anti-AChE enzyme inhibition was found to be 47.83 ± 0.21% with an IC_50_ of 4.76 ± 0.01 µg/mL. Additionally, SB-CuO-NPs possess anti-arthritic activity as anti-protein denaturation with inhibition (%) = 38.27 ± 0.17 and as an anti-proteinase enzyme (inhibition (%) = 35.57 ± 0.17% and IC_50_ = 7.13 ± 0.03 µg/mL). The results of anti-inflammatory effects express that SB-CuO-NPs possess the capability to act as an anti-inflammatory agent with IC_50_ values of 6.49 ± 0.07, 4.83 ± 0.02, and 8.25 ± 0.03 µg/mL against COX-1, COX-2, and 5-LOX enzymes, respectively. Finally, the SB-CuO-NPs exhibited enhanced cytotoxic activity against HepG-2, Caco-2, and A549 cancer cells with IC_50_ values of 18.68 ± 0.83, 17.08 ± 1.30, and 13.87 ± 2.25 µg/mL, respectively. Largely, the SB-CuO-NPs possess better biological applications than the SB.

Further studies are recommended to be performed in the future to reveal the biological efficiency of CuO-NPs synthesized using Schiff base as a reducing agent against chronic diseases induced chemically in experimental animals (in vivo study).

## Figures and Tables

**Table 1:** The in vitro antioxidant and radicals scavenging activities of the SB-CuO-NPs, SB, and ascorbic acid.

Activities		Units	SB	SB-CuO-NPs	Ascorbic Acid
Antioxidant activity	TAC	mg gallic acid/g	65.41 ± 0.19	76.53 ± 0.22	83.75 ± 0.13
	IRP	µg/mL	47.66 ± 0.19	58.78 ± 0.22	66.00 ± 0.13
Scavenging activity	DPPH	Inhib. (%)	44.54 ± 0.18	54.93 ± 0.21	61.68 ± 0.12
		IC_50_ (µg/mL)	6.24 ± 0.01	5.06 ± 0.01	4.50 ± 0.03
	ABTS	Inhib. (%)	48.29 ± 0.18	58.68 ± 0.21	65.43 ± 0.12
		IC_50_ (µg/mL)	5.40 ± 0.06	4.45 ± 0.05	3.99 ± 0.04
	NO	Inhib. (%)	36.79 ± 0.18	47.18 ± 0.21	53.93 ± 0.12
		IC_50_ (µg/mL)	7.69 ± 0.04	5.99 ± 0.03	5.24 ± 0.02

The values were calculated from n = 3 and given as mean ± SE.

**Table 2:** The in vitro anti-diabetic and anti-Alzheimer activities of the SB-CuO-NPs, SB, and standard reference.

Activities		Units	SB	SB-CuO-NPs	Standard Reference
Anti-diabetic activity	α-Amylase	Inhib. (%)	38.63 ± 0.14	51.03 ± 0.18	54.96 ± 0.10 Acarbose
		IC_50_ (µg/mL)	5.15 ± 0.02	3.90 ± 0.02	3.62 ± 0.03 Acarbose
	α-Glucosidase	Inhib. (%)	28.38 ± 0.14	42.82 ± 0.19	44.71 ± 0.10 Acarbose
		IC_50_ (µg/mL)	3.69 ± 0.06	2.45 ± 0.04	2.35 ± 0.02 Acarbose
Anti-Alzheimer activity	Acetylcholinesterase (AChE)	Inhib. (%)	32.64 ± 0.16	47.83 ± 0.21	51.42 ± 0.12 Donepezil
		IC_50_ (µg/mL)	6.97 ± 0.02	4.76 ± 0.01	4.42 ± 0.02 Donepezil

The values were calculated from n = 3 and given as mean ± SE.

**Table 3:** The in vitro anti-arthritic and anti-inflammatory activities of the SB-CuO-NPs, SB, and standard reference.

Activities		Units	SB	SB-CuO-NPs	Standard Reference
Anti-arthritic activity	Protein Denaturation	Inhib. (%)	26.11 ± 0.13	38.27 ± 0.17	41.14 ± 0.09 Diclofenac Sodium
	Proteinase	Inhib. (%)	23.41 ± 0.13	35.57 ± 0.17	38.44 ± 0.09 Diclofenac Sodium
		IC_50_ (µg/mL)	10.84 ± 0.06	7.13 ± 0.03	6.60 ± 0.02 Diclofenac Sodium
Anti-inflammatory activity	COX-1	Inhib. (%)	31.61 ± 0.18	48.02 ± 0.23	51.89 ± 0.13 Indomethacin
		IC_50_ (µg/mL)	9.86 ± 0.11	6.49 ± 0.07	6.01 ± 0.02 Indomethacin
	COX-2	Inhib. (%)	33.86 ± 0.18	50.27 ± 0.23	54.14 ± 0.13 Indomethacin
		IC_50_ (µg/mL)	7.15 ± 0.01	4.83 ± 0.02	4.47 ± 0.03 Indomethacin
	5-LOX	Inhib. (%)	26.71 ± 0.18	43.12 ± 0.23	46.99 ± 0.13 Zileuton
		IC_50_ (µg/mL)	13.33 ± 0.07	8.25 ± 0.03	7.57 ± 0.03 Zileuton

The values were calculated from n = 3 and given as mean ± SE.

**Table 4:** The in vitro cytotoxic activities of the SB-CuO-NPs, SB, and doxorubicin against human hepatocellular carcinoma (HepG-2), colon (Caco-2), lung (A549) cancer cell lines, and normal human fibroblast cells (BJ-1).

Activities		Units	SB	SB-CuO-NPs	Doxorubicin
Median Inhibitory Concentration (IC_50_)	HepG-2	IC_50_ (µg/mL)	127.16 ± 3.13	18.68 ± 0.83	15.49 ± 1.98
	Caco-2	IC_50_ (µg/mL)	115.16 ± 3.25	17.08 ± 1.30	11.13 ± 1.09
	A549	IC_50_ (µg/mL)	110.37 ± 2.49	13.87 ± 2.25	14.36 ± 1.12
	BJ-1	IC_50_ (µg/mL)	167.65 ± 2.22	24.23 ± 0.76	22.00 ± 1.51

The values were calculated from n = 3 and given as mean ± SE.

**Table 5:** The enzymatic assay values after the treatment of the SB-CuO-NPs, SB, and standard reference with human hepatocellular carcinoma (HepG-2), colon (Caco-2), lung (A549) cancer cell lines, and normal human fibroblast cells (BJ-1).

Activities		Units	DMSO	SB	SB-CuO-NPs	Doxorubicin
HepG-2	Caspase-3	Pg/mL	94.60 ± 0.74	165.55 ± 1.29	291.00 ± 1.34	306.04 ± 1.23
	Bcl-2	ng/mL	12.82 ± 0.17	7.32 ± 0.10	4.17 ± 0.05	4.00 ± 0.02
Caco-2	Caspase-3	Pg/mL	82.44 ± 0.12	144.28 ± 0.22	252.31 ± 0.30	265.13 ± 0.12
	Bcl-2	ng/mL	6.60 ± 0.09	3.77 ± 0.05	2.14 ± 0.01	2.04 ± 0.01
A549	Caspase-3	Pg/mL	77.11 ± 0.10	134.94 ± 0.18	236.30 ± 0.22	247.96 ± 0.08
	Bcl-2	ng/mL	14.46 ± 0.05	8.26 ± 0.03	4.72 ± 0.02	4.51 ± 0.01
BJ-1	Caspase-3	Pg/mL	62.32 ± 0.10	109.07 ± 0.18	191.03 ± 0.22	200.43 ± 0.08
	Bcl-2	ng/mL	8.71 ± 0.05	4.98 ± 0.03	2.85 ± 0.02	2.72 ± 0.01

The values were calculated from n = 3 and given as mean ± SE.

## Data Availability

Data is contained within the article or Supplementary Materials.

## Supplementary Materials

The following supporting information can be downloaded at: https://www.mdpi.com/article/10.3390/pharmaceutics17020180/s1, Table S1: The statistical correlations among the different in vitro biological activities of SB-CuO-NPs and SB at equal concentrations (100 µg/mL); Table S2: Data of the cytotoxic activity of the SB-CuO-NPs against human hepatocellular carcinoma (HepG-2) cell line compared to the Schiff base (SB) and Doxorubicin as standard; Table S3: Data of the cytotoxic activity of the SB-CuO-NPs against human colon cancer (Caco-2) cell line compared to the Schiff base (SB) and Doxorubicin as standard; Table S4: Data of the cytotoxic activity of SB-CuO-NPs against human lung cancer (A549) cell lines cell line compared to the Schiff base (SB) and Doxorubicin as standard; Table S5: Data of the cytotoxic activity of the SB-CuO-NPs against human normal human fibroblast (BJ-1) cell line compared to the Schiff base (SB) and Doxorubicin as standard; Preparation of the Schiff base (SB); 1H-NMR data of the Schiff base (SB) and biological methods.
