# Supplementary material for: Assessment of the In Vitro Biological Activities of Schiff Base-Synthesized Copper Oxide Nanoparticles as an Anti-Diabetic, Anti-Alzheimer, and Anti-Cancer Agent
Source: Pharmaceutics. 2025 Feb 1;17(2):180. doi: 10.3390/pharmaceutics17020180 (PMC11859031; doi:10.3390/pharmaceutics17020180)
Supplement: Supplementary file 1 [file pharmaceutics-17-00180-s001.zip › pharmaceutics-3434493-supplementary.pdf]

Synthesis of 3-(((5-chloro-3-methyl-1-phenyl-1*H*-pyrazol-4-yl)methylene)hydrazono) indolin-2-one (SB).

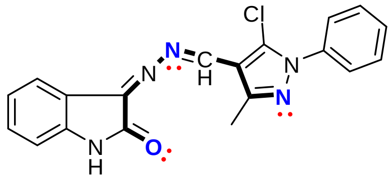

Schiff base (SB)

A mixture of 3-hydrazonoindolin-2-one (**1**) (0.01 mol, 1.61 g) with 5-chloro-3-methyl-1-phenyl-1*H*-pyrazole-4-carbaldehyde (**2**) (0.01 mol, 2.20 g) and a catalytic amount of glacial acetic acid (0.5 mL) in absolute ethanol (25 mL) was refluxed for four hours and then left to cool. The solid product was filtered of, dried, and finally recrystallized from ethanol to afford Schiff base (SB).

Orange crystals; M.p.: 268-270 °C.

^{1}H NMR (DMSO-d_6, 500 MHz, δ ppm) 2.58 (s, 3H, CH_3), 6.89 (d, 1H, *J* = 8.3 Hz, isatin), 7.03 (t, 1H, isatin), 7.39 (t, 1H, isatin), 7.52-7.65 (m, 5H, aromatic-H), 8.15 (d, 1H, *J* = 8.3 Hz, isatin), 8.65 (s, 1H, -CH=N-, azomethine), 10.85 (s, 1H, NH-isatin).

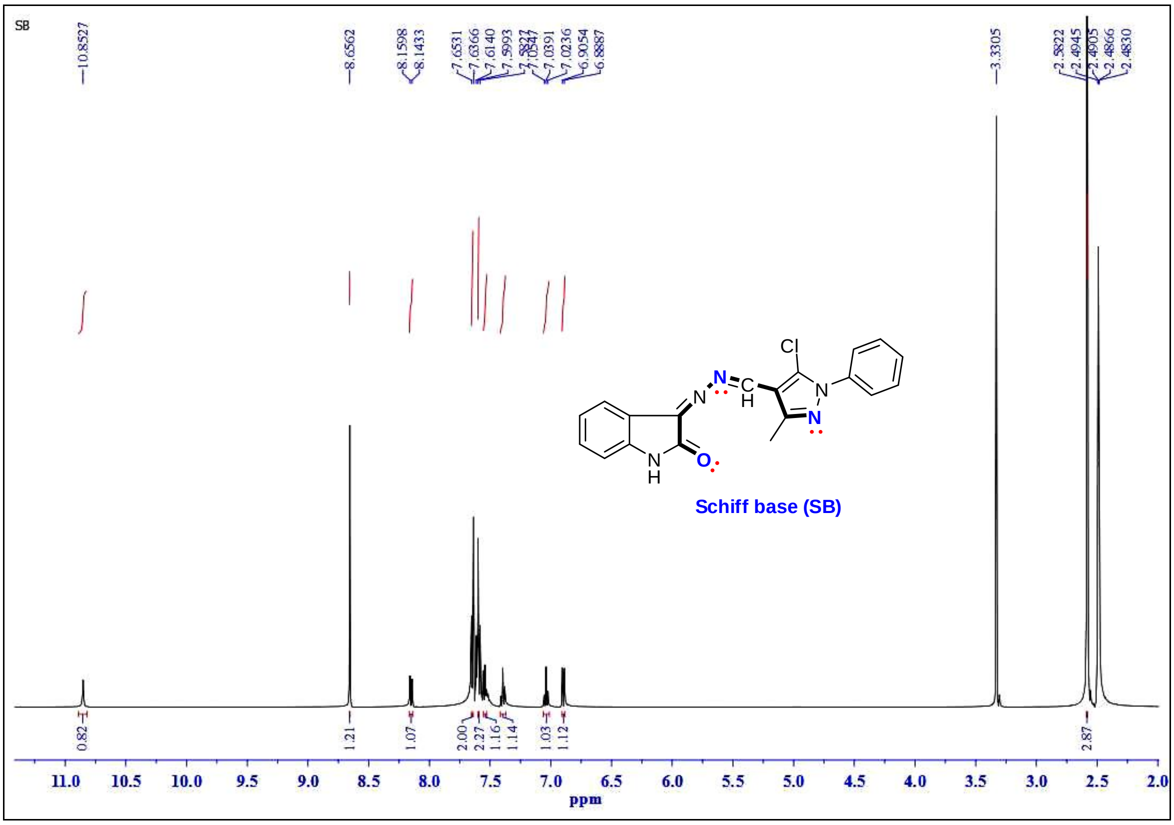

^{1}H-NMR spectrum of SB

Table S1: The statistical correlations among the different *in vitro* biological activities of SB-CuO-NPs and SB at equal concentrations (100 μg/mL)

		Antioxidant		Scavenging		Anti-diabetic		Anti-Alzheimer	Anti-arthritic		Anti-inflammatory		
		**TAC**	**IRP**	**DPPH**	**ABTS**	**α-Amy**	**α-gluc**	**AChE**	**Protein Denaturation**	**Proteinase**	**COX-1**	**COX-2**	**5-LOX**
**Antioxidant**	**TAC**	-	0.000**	0.000**	0.000**	0.000**	0.000**	0.000**	0.000**	0.000**	0.000**	0.000**	0.000**
	**IRP**	0.000**	-	0.000**	0.000**	0.000**	0.000**	0.000**	0.000**	0.000**	0.000**	0.000**	0.000**
**Scavenging**	**DPPH**	0.000**	0.000**	-	0.000**	0.000**	0.000**	0.000**	0.000**	0.000**	0.000**	0.000**	0.000**
	**ABTS**	0.000**	0.000**	0.000**	-	0.000**	0.000**	0.000**	0.000**	0.000**	0.000**	0.000**	0.000**
**Anti-diabetic**	**α-amy**	0.000**	0.000**	0.128	0.000**	-	0.000**	0.000**	0.000**	0.000**	0.000**	0.000**	0.000**
	**α-gluc**	0.000**	0.000**	0.177	0.000**	0.000**	-	0.000**	0.000**	0.000**	0.000**	0.000**	0.000**
**Anti-Alzheimer**	**AChE**	0.000**	0.000**	-0.363	0.000**	0.000**	0.000**	-	0.000**	0.000**	0.000**	0.000**	0.000**
**Anti-arthritic**	**Protein Denaturation**	0.000**	0.000**	0.000**	0.000**	0.000**	0.000**	0.000**	-	0.000**	0.000**	0.000**	0.000**
	**Proteinase**	0.000**	0.000**	0.000**	0.000**	0.000**	0.000**	0.000**	0.000**	-	0.000**	0.000**	0.000**
**Anti-inflammatory**	**COX-1**	0.000**	0.000**	0.000**	0.000**	0.000**	0.000**	0.000**	0.000**	0.000**	-	0.000**	0.000**
	**COX-2**	0.000**	0.000**	0.000**	0.000**	0.000**	0.000**	0.000**	0.000**	0.000**	0.000**	-	0.000**
	**5-LOX**	0.000**	0.000**	0.000**	0.000**	0.000**	0.000**	0.000**	0.000**	0.000**	0.000**	0.000**	-

** indicates a positive correlation ($p \leq 0.01$).

Table S2: Data of the cytotoxic activity of the SB-CuO-NPs against human hepatocellular carcinoma (HepG-2) cell line compared to the Schiff base (SB) and Doxorubicin as standard.

Schiff base (SB)						
**Conc. (μg/mL)**	**0.00**	**31.13**	**62.50**	**125.00**	**250.00**	**500.00**
Mean OD	0.48	0.40	0.35	0.24	0.17	0.03
Viability %	98.85	87.58	74.58	53.10	40.19	19.30
**IC_{50} (μg/mL)**	**127.16 ± 3.13**					
SB-CuO-NPs						
**Conc. (μg/mL)**	**0.00**	**6.25**	**12.50**	**25.00**	**50.00**	**100.00**
MeanOD	0.45	0.38	0.33	0.22	0.16	0.02
Viability %	100.00	81.64	67.81	36.22	23.99	4.22
**IC_{50} (μg/mL)**	**18.68 ± 0.83**					
**DOX**						
**Conc. (μg/mL)**	**0.00**	**6.25**	**12.50**	**25.00**	**50.00**	**100.00**
MeanOD	0.48	0.40	0.30	0.23	0.16	0.08
Viability %	100.00	74.37	59.01	34.34	18.09	9.17
**IC_{50} (μg/mL)**	**15.49 ± 1.98**					

Table S3: Data of the cytotoxic activity of the SB-CuO-NPs against human colon cancer (Caco-2) cell line compared to the Schiff base (SB) and Doxorubicin as standard.

Schiff base (SB)						
**Conc.** (μg/mL)	0.00	31.13	62.50	125.00	250.00	500.00
Mean OD	0.39	0.32	0.26	0.16	0.10	0.07
Viability %	98.85	86.03	72.20	48.61	38.38	18.61
**IC_{50} (μg/mL)**	**115.16 ± 3.25**					
SB-CuO-NPs						
**Conc.** (μg/mL)	**0.00**	**6.25**	**12.50**	**25.00**	**50.00**	**100.00**
MeanOD	0.42	0.34	0.29	0.18	0.11	0.07
Viability %	100.00	79.02	63.12	35.54	26.63	5.74
**IC_{50} (μg/mL)**	**17.08 ± 1.30**					
**DOX**						
**Conc.** (μg/mL)	**0.00**	**6.25**	**12.50**	**25.00**	**50.00**	**100.00**
MeanOD	0.41	0.35	0.25	0.16	0.18	0.04
Viability %	100.00	70.23	35.18	5.64	4.48	1.30
**IC_{50} (μg/mL)**	**11.13 ± 1.09**					

Table S4: Data of the cytotoxic activity of SB-CuO-NPs against human lung cancer (A549) cell lines cell line compared to the Schiff base (SB) and Doxorubicin as standard.

Schiff base (SB)						
**Conc.** (μg/mL)	0.00	31.13	62.50	125.00	250.00	500.00
Mean OD	0.49	0.43	0.38	0.28	0.22	0.09
Viability %	98.85	86.18	70.35	44.76	38.53	18.75
**IC_{50} (μg/mL)**	**110.37 ± 2.49**					
SB-CuO-NPs						
**Conc.** (μg/mL)	**0.00**	**6.25**	**12.50**	**25.00**	**50.00**	**100.00**
MeanOD	0.51	0.44	0.39	0.29	0.22	0.09
Viability %	100.00	71.55	49.25	31.70	26.01	5.50
**IC_{50} (μg/mL)**	**13.87 ± 2.25**					
**DOX**						
**Conc.** (μg/mL)	**0.00**	**6.25**	**12.50**	**25.00**	**50.00**	**100.00**
MeanOD	0.27	0.23	0.17	0.13	0.09	0.80
Viability %	100.00	77.37	51.01	24.34	12.09	5.17
**IC_{50} (μg/mL)**	**14.36 ± 1.12**					

Table S5: Data of the cytotoxic activity of the SB-CuO-NPs against human normal human fibroblast (BJ-1) cell line compared to the Schiff base (SB) and Doxorubicin as standard.

Schiff base (SB)						
**Conc.** (μg/mL)	**0.00**	**31.13**	**62.50**	**125.00**	**250.00**	**500.00**
Mean OD	0.35	0.29	0.27	0.18	0.11	0.07
Viability %	98.85	90.93	80.93	62.69	47.25	19.13
**IC_{50} (μg/mL)**	**167.65 ± 2.22**					
**SB-CuO-NPs**						
**Conc.** (μg/mL)	**0.00**	**6.25**	**12.50**	**25.00**	**50.00**	**100.00**
MeanOD	0.36	0.31	0.28	0.19	0.11	0.07
Viability %	100.00	86.37	74.17	51.07	35.06	5.88
**IC_{50} (μg/mL)**	**24.23 ± 0.76**					
**DOX**						
**Conc.** (μg/mL)	**0.00**	**6.25**	**12.50**	**25.00**	**50.00**	**100.00**
MeanOD	0.27	0.23	0.17	0.13	0.09	0.80
Viability %	100.00	87.37	68.01	42.34	20.09	13.17
**IC_{50} (μg/mL)**	**22.00 ± 1.51**					

Material and Methods

1. Preparation of SB-CuO-NPs

The copper oxide nanoparticles (CuO-NPs) were biosynthesized using a concentrated anhydrous copper sulfate solution (1 mM) as a metal precursor based on the method suggested by **Rani Verma and Khan (2019)**. Each precursor was dissolved in 100 ml of deionized water to form a 0.1 M concentration. The Schiff base (SB) was mixed with the metal solution in a ratio of 4:1. The **SB-CuO-NPs** was monitored by a color change. They were dried at 80 °C after centrifuging the colloidal solution at 4000 rpm for 15 min.

2. Characterization of the SB-CuO-NPs

The spectra of the **SB-CuO-NPs** were characterized using a Shimadzu UV-VIS recording spectrophotometer UV-240 after diluting the samples (10-fold) with deionized water at λ 200 - 800 nm. Their surface zeta potentials were measured using the laser zeta meter (Malvern Zetasizer 2000, Malvern, Worcestershire, UK) following the method demonstrated by **Meléndrez and colleagues (2010)** who suggested that the particles could be neutral (with a zeta potential between -10 and +10 mV), strongly cationic (greater than +30 mV) or strongly anionic (less than -30 mV). Moreover, their average hydrodynamic sizes were determined using the laser diffraction method with multiple scattering techniques employing dynamic light scattering (DLS) (Malvern Zetasizer Nano ZS, Malvern Instruments Ltd., Malvern, United Kingdom) **(Murdock *et al.*, 2008)**.

3. *In vitro* biological activities

All biological activities were assessed in the prepared samples at equal concentrations (100 µg/mL). All assays were carried out in triplicate.

3.1. Antioxidant activity

The total antioxidant capacity (TAC) was determined by analyzing the green phosphate/Mo^{5+} complex at a wavelength (λ) of 695 nm, following the procedure described by **Prieto *et al.*** Samples (at each concentration) were mixed with a reagent solution containing 0.3 N sulfuric acid, 28 mM sodium phosphate, and 4 mM

ammonium molybdate. Methanol (80%) was used in place of the sample for the blank. The tubes were sealed and incubated in a boiling water bath for 90 minutes. After cooling to room temperature, the absorbance was measured at 695 nm against the blank. Ascorbic acid was used at the same concentrations as a standard. The antioxidant capacity was expressed as mg gallic acid equivalent per gram weight.

The iron reducing power was determined as μg/mL using the method proposed by **Oyaizu (1986)**. In brief, 1ml of the tested sample (at each concentration) was combined with 1mL of 200mM sodium phosphate buffer (pH 6.6) and 1mL of 1% potassium ferricyanide. The mixture was then incubated at 50°C for 20 minutes, followed by the addition of 1mL of trichloroacetic acid (10%). After centrifugation at 2000rpm for 10 minutes, the upper layer solution (2.5 mL) was mixed with 2.5 mL of double deionized water and 1mL of fresh ferric chloride (0.1%). The absorbance was measured at 700nm against a blank prepared without the sample. Ascorbic acid was used at the same concentrations as a standard. A high absorbance at 700nm indicates a higher reducing power in the reaction mixture.

3.2. Scavenging activity

The 1,1-Diphenyl-2-picryl-hydrazyl (DPPH) radical scavenging activities were evaluated using the method described by **Rahman *et al.*** An antioxidant substance capable of donating a hydrogen atom to a solution containing DPPH- can reduce the stable free radical, causing the solution to change color from violet to pale yellow. The remaining DPPH- radical was quantified by measuring the intensity of a light-purple colored DPPH methanol solution in the visible range at 518 nm using a spectroscopic method. Two milliliters of a DPPH solution (100 μM) in ethanol were mixed with 2 mL of the sample (at each concentration). The reaction mixture for each concentration was thoroughly vortexed and then incubated in the dark at room temperature for 30 minutes. The absorbance was then measured spectrophotometrically at 518 nm against a blank (ethanol). For the control, 2 mL of ethanol was added instead of the sample and run simultaneously with the test. Ascorbic acid was used at the same concentrations as a positive control. Percent

inhibition of the DPPH free radical was calculated. The median inhibitory concentration (IC_{50}) for each tested compound was calculated using a series of concentrations (0, 0.75, 1.56, 3.125, 6.25, 12.5, 25, 50, and 100 μg/mL).

The procedure for the 2,2'-azinobis-(3-ethylbenzothiazoline-6-sulfonic acid) (ABTS) assay followed the method suggested by **Arnao *et al.*** Stock solutions included ABTS solution (7 mM) and potassium persulfate solution (2.4 mM). The working solution was prepared by mixing the two stock solutions in equal quantities and allowing them to react at room temperature in a dark place for 14 hours. The solution was then diluted by mixing 1 mL of ABTS solution with 60 mL of methanol to obtain an absorbance of 0.706 ± 0.01 units at 734 nm using a spectrophotometer. Fresh ABTS solution was prepared for each assay. The tested samples (at each concentration) were allowed to react with 1 mL of the ABTS solution, and the absorbance was taken at 734 nm after 7 minutes using a spectrophotometer. The ABTS scavenging capacities of the samples were compared with that of ascorbic acid (at the same concentrations).

3.3. Anti-diabetic activity

This assay involved calculating the inhibition percentage (%) of α-amylase enzyme using method based on the technique demonstrated by Wickramaratne *et al*. with Acarbose as the standard drug. During the assay, 0.5 ml of the test solution was combined with 0.5 ml of α-amylase solution (0.5 mg/ml) and buffer (Na_2HPO_4/NaH_2PO_4 (0.02 M), NaCl (0.006 M) at pH 6.9) to create concentrations ranging from 25 to 800 μg/mL. The mixture was then left at room temperature for 10 minutes before adding 200 μL of starch solution (1% in water (w/v) buffer (Na_2HPO_4/NaH_2PO_4 (0.02 M), NaCl (0.006 M) at pH 6.9)). The reaction was stopped by adding 200 μL of DNSA (coloring) reagent (12 g of sodium potassium tartrate tetrahydrate in 8.0 mL of 2 M NaOH and 20 mL of 96 mM of DNSA solution). The test tubes were then placed in a boiling water bath (100 °C) for 10 minutes and the mixture was cooled to room temperature and diluted with 5 mL of distilled water.

The absorbance was measured at 540 nm using a UV-Visible spectrophotometer. The IC_{50} of each tested sample was calculated by plotting a curve using a series of sample concentrations against the percent of α-amylase inhibition.

The inhibition percentage (%) of the α-glucosidase enzyme was determined using the method proposed by Pistia-Brueggeman and Hollingsworth with Acarbose as the standard drug. Five μL of the α-glucosidase solution (10 units mL^{-1}, 0.1moll^{-1} potassium phosphate buffer, pH 6.8) was pre-mixed with 10 μL of the sample solution at different concentrations (in 10% DMSO) in 620 μL of 0.1 moll^{-1} potassium phosphate buffer (pH 6.8). After incubation at 37.5 °C for 20 minutes, 10 μL of p-nitro phenyl glucopyranoside (pNPG, 10 mmoll^{-1}) as a substrate was added to the mixture to start the reaction. The reaction mixture was then incubated at 37.5°C for 30 minutes, followed by the addition of 650 μL of 1 moll^{-1} Na_2CO_3 solution to terminate the reaction. The amount of released product (p-nitro phenol) was measured at 410 nm using a UV spectrometer (UV-2550, Shimadzu, Japan) to estimate the enzymatic activity. The inhibition assay was performed in triplicate for all tests. The IC_{50} of each tested sample was calculated by plotting a curve using a series of sample concentrations against the percent of α-glucosidase inhibition.

3.4. Anti-Alzheimer's activity

In this study, we assessed the inhibition percentage of the acetylcholinesterase (AChE) enzyme using Ellman's method with donepezil as the standard drug. For each run, 5 μL of Acetylthiocholine (ATCh) at a concentration of 0.5 mM, 5 μL of 5,5′-dithiobis-2-nitrobenzoic acid (DTNB) at a concentration of 0.03 mM, and 5 μL of each sample (at each concentration) were added to a flat bottom 96-well plate. The mixture was then incubated for 10 minutes at 30 °C. After incubation, 5 μL of AChE at a concentration of 0.3 U/mL was added to start the reaction, and the absorbance was measured at 412nm. A control run was also performed, which included all the components except for the test sample. The median inhibitory concentration (IC_{50}) of each tested sample was calculated by plotting a curve using a series of sample concentrations against the percent of AChE inhibition.

3.5. Anti-arthritic activity

In the anti-arthritic activity study, this assay involved determining the percentage of protein denaturation **(Das and Sureshkumar, 2016)** and proteinase **(Oyedapo and Famurewa, 1995)** inhibition using diclofenac sodium as the standard non-steroidal anti-inflammatory drug, as prepared according to **Meera *et al.*** The protein denaturation percentage was measured by mixing 0.5mL of the test control solution, prepared by combining 0.45 mL of bovine serum albumin (BSA) (5% w/v aqueous solution) with 0.05 mL of distilled water. Then, 0.05 mL of the test solution was added to 0.45 mL of distilled water to form the product control (0.5 mL). The different samples (test solution) and diclofenac sodium (standard) were used. The pH value in all prepared solutions was adjusted to 6.3 using HCl (1N). All the samples were incubated at 37 ^{o}C for 20 min, and the temperature was then increased to 57 ^{o}C, maintaining the samples at that degree for 3 min. After cooling, 2.5 mL of phosphate buffer was added to the prepared solutions. The absorbance was determined at 416 nm using a UV-Visible spectrophotometer. The percentage of protein denaturation inhibition can be calculated. Proteinase inhibitory activity was assessed by combining the test sample (1 mL) with a reaction mixture containing 0.06 mg trypsin dissolved in 1 mL of 20 mM Tris HCl buffer (pH 7.4). The mixture was then incubated for 5 minutes at 37°C, followed by the addition of 1 mL of casein (0.8% w/v). After an additional 20 minutes of incubation, 2 mL of perchloric acid (70%) was added to stop the reaction. The cloudy suspension was then centrifuged, and the absorbance of the supernatant was measured at 210 nm against buffer as the blank. The percentage of proteinase inhibitory activity was then calculated.

3.6. Anti-inflammatory activity

In vitro anti-inflammatory evaluation was performed through inhibition of two isoenzymes cyclooxygenase COX-1 and COX-2 (ovine/human), along with 5-LOX enzyme (human recombinant). COX-1 and COX-2 inhibition assay has been performed by means of COX-1 and COX-2 kit (Cayman, No.: 560131), where different known concentrations of the tested compounds were added separately to a

mixture of 10 µL of COX-1 or COX-2 and 0.1 M HCl buffer, left for incubation at room temperature for 10 min. After that, 10µL of arachidonic acid, fifty µL HCl and Ellman's reagent have been added. The absorbance has been determined at UV-410 nm alongside blank; IC_{50} has been determined via linear regression.

5-Lipoxygenase inhibition assay was carried out by 5-LOX kit (No. 437996, Sigma-Aldrich), where different concentrations of the tested compounds were added to 90 µL from 5-LOX, 100 µL of de chromogen, then 10 µL from arachidonic acid was added and shaken for 10 min, the absorbance has been determined at UV-490 nm compared to blank. IC50 has been calculated through linear regression.

Statistical analysis

The Statistical Package for the Social Sciences (SPSS) for Windows, version 11.0, was used to conduct statistical analysis, which included one-way analysis of variance (ANOVA) to assess both positive and negative correlations between in vitro biological activities to determine significant relationships. A "p" value less than 0.05 was used as the significance criterion. Each sample was analyzed in triplicate at a concentration of 100 µg/mL.

3.7. Cytotoxic activity and Enzymatic Assay

It will be assayed against human hepatocellular (HepG-2), colon carcinoma (Caco-2), and lung cancer (A549) cells and compared to normal human fibroblast (BJ-1) using 3-(4,5-dimethythiazol-2-yl)-2,5-diphenyl tetrazolium bromide (MTT) assay by determining the optical density (OD) at 570 nm according to the method suggested by **Vichai and Kirtikara**. The cells were dispensed in a 96-well sterile microplate (3×10^4 cells/ well), followed by their incubation at 37 °C with a series of different concentrations of 10 µL of each compound or doxorubicin (positive control, in DMSO) for 48 h in a serum free medium prior to the MTT assay. Subsequently, the media were carefully removed, and 40 µL of MTT (2.5 mg/mL) was added to each well and then incubated for an additional 4 h. Purple formazan dye crystals were solubilized by the addition of 200 µL of DMSO. The absorbance was measured at 570 nm using a SpectraMax Paradigm Multi-Mode microplate reader. The relative cell

viability was expressed as the mean percentage of viable cells relative to the untreated control cells. All experiments were conducted in triplicate. Percent of the cell-growth inhibition (%) and the IC_{50} was calculated using IC_{50} calculation software. Activities of caspase-3 were measured by enzyme-linked immunosorbent assay (ELISA) using the Invitrogen caspase-3 (Active) (human) ELISA kit (96 tests) from Invitrogen Corporation, following the manufacturer's instructions. Activities of Bcl-2 were measured using the Invitrogen Zymed Bcl-2 ELISA Kit (96 tests) from Invitrogen Corporation, following the manufacturer's instructions.
